# Binary and ternary cocrystals of sulfa drug acetazolamide with pyridine carboxamides and cyclic amides

**DOI:** 10.1107/S2052252516000543

**Published:** 2016-02-25

**Authors:** Geetha Bolla, Ashwini Nangia

**Affiliations:** a School of Chemistry, University of Hyderabad, Prof. C. R. Rao Road, Central University PO, Hyderabad 500 046, India

**Keywords:** crystal engineering, co-crystals, crystal design, hydrogen bonding, pharmaceutical solids

## Abstract

A novel design strategy for cocrystals of a sulfonamide drug with pyridine carboxamides and cyclic amides is developed based on synthon identification as well as size and shape match of coformers. Binary adducts of acetazolamide (ACZ) with lactams (valerolactam and caprolactam, VLM, CPR), cyclic amides (2-pyridone, labeled as 2HP and its derivatives MeHP, OMeHP) and pyridine amides (nicotinamide and picolinamide, NAM, PAM) were obtained by manual grinding, and their single crystals by solution crystallization. The heterosynthons in the binary cocrystals of ACZ with these coformers suggested a ternary combination for ACZ with pyridone and nicotinamide. Novel supramolecular synthons of ACZ with lactams and pyridine carboxamides are reported together with binary and ternary cocrystals for a sulfonamide drug. This crystal engineering study resulted in the first ternary cocrystal of acetazolamide with amide coformers, ACZ–NAM–2HP (1:1:1).

## Abstract 2

The first report of a ternary cocrystal acetazolamide–nicotinamide–pyridone (1:1:1) for a sulfonamide drug with amide coformers.

## 1. Introduction   

**Figure 1:**
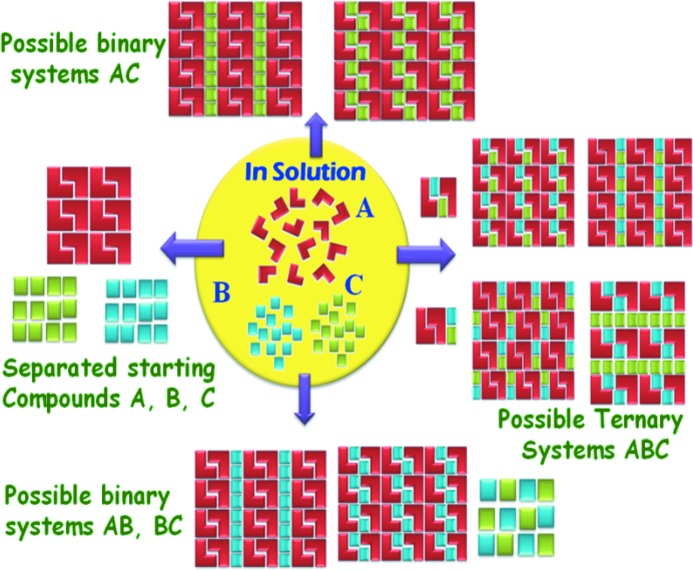
Multiple possibilities of solid forms during cocrystallization to give single, binary and ternary products.

Hydrogen bonding is the key adhesive to construct supramolecular synthons for the design of crystalline architectures by using multiple functional groups (Desiraju, 1995 ▸). From a crystal engineering perspective, binary and ternary adducts are formed due to robust heterosynthons in the cocrystal, compared with homosynthons in the constituent molecules (Walsh *et al.*, 2003 ▸). It has been shown over more than a decade that crystal engineering of multi-component phases offers rational approaches to systematically tune the physicochemical and pharmacokinetic properties of active pharmaceutical ingredients (APIs, Fig. 1 ▸). The matching of functional groups and supramolecular synthons together with size and shape factors of molecules offers an approach to assemble three different molecules in the same crystal lattice (Tothadi & Desiraju, 2013 ▸, 2014 ▸; Chakraborty *et al.*, 2014 ▸; Aakeröy *et al.*, 2001 ▸, 2005 ▸; Seaton *et al.*, 2013 ▸; Aitipamula *et al.*, 2013 ▸). Ternary cocrystals are relatively less studied and the sulfonamide group is a ‘structural gap’, even as SO_2_NH_2_ is the key functional group in the most populated sulfa drugs category. These considerations encouraged us to systematically study binary and ternary cocrystals of the sulfonamide group (Bolla *et al.*, 2015 ▸). The assembly of three different molecular components in the same crystal lattice is challenging because it hinges on a balance of intermolecular interaction strengths, chemical recognition, geometric fit and overall shape complementarity (Tothadi & Desiraju, 2013 ▸). There is more than one possible outcome of a three-component cocrystallization; it may result in one of the components, its solvates or hydrate, a new polymorph of the molecule, binary systems, starting materials, or the ternary product (Fig. 1 ▸).

Recent success in the deliberate construction of ternary cocrystals (Bolla & Nangia, 2015 ▸) and our work on binary sulfonamide cocrystals (Bolla *et al.*, 2014 ▸, 2015 ▸) served as the background for the present study. The Cambridge Structural Database (CSD version 5.36, May 2105 update) contains about 75 X-ray crystal structures of ternary systems. Recently, we reported the assembly of ternary components using amides and the sulfonamide group along with a carboxylic acid (Bolla & Nangia, 2015 ▸). In the present work, the sulfonamide and acetamide groups of acetazolamide are the starting point to demonstrate the sulfonamide–lactam supramolecular synthon for the assembly of ternary systems.

**Figure 2:**
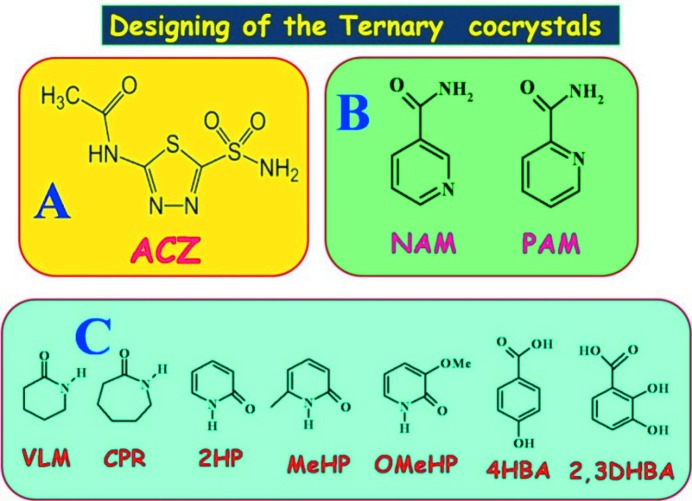
Chemical structures of ACZ as **A**, coformers pyridine carboxamides and *syn*-amides, aromatic COOH compounds as **B** and **C** used in cocrystallization.

Acetazolamide, 5-acetamido-1,3,4-thiadiazole-2-sulfon­amide (Fig. 2 ▸), is an antiepileptic, diuretic drug for respiratory diseases (Arenas-García *et al.*, 2010 ▸, 2012 ▸; Grecu *et al.*, 2014 ▸). It is also used to prevent the symptoms of altitude sickness as this medication decreases headache, tiredness, nausea, dizziness and shortness of breath at high altitudes. This drug is also used to treat open-angle glaucoma by reducing the amount of fluid that can build up in the eye. The aqueous solubility of ACZ (0.72 mg ml^−1^ in water at 25°C) is low, has poor permeability and, according to the Biopharmaceutics Drug Disposition Classification System (BDDCS), ACZ belongs to the low solubility and poor permeability Class IV category (Granero *et al.*, 2008 ▸; Benet, 2010 ▸). ACZ is administered as a 250 mg dose according to the World Health Organization’s list (WHO) of essential medicines, a list of the most important medications needed in a basic healthcare system (WHO list dated April 2013, http://apps.who.Int/iris/bitstream/10665/93142/1/EML_18_eng.pdf?ua = 1, accessed 15 Nov. 2015). Two polymorphs of ACZ, forms (I) and (II), and cocrystals with 4-hydroxybenzoic acid, nicotinamide, 4-hydroxybenzamide, picolinamide, 2,3-dihydroxy benzoic acid, and a few inorganic coordination complexes with Ni, Cu, Zn are reported (Umeda *et al.*, 1985 ▸; Baraldi *et al.*, 2009 ▸; Arenas-García *et al.*, 2010 ▸, 2012 ▸; Ferrer *et al.*, 1989 ▸, 1990 ▸; Hartmann & Vahrenkamp, 1991 ▸).

The work plan described in this paper was to first prepare binary cocrystals of ACZ with nicotinamide (ACZ–NAM, 1:1), valerolactam (ACZ–VLM, 1:2), caprolactam (ACZ–CPR hydrate, 1:1:1), 2-pyridone (ACZ–2HP, 1:1 and 1:2), 6-methyl-2-pyridone (ACZ–MeHP, 1:1) and 3-methoxy-2-pyridone (ACZ–OMeHP, 1:2), with the idea of assessing the hydrogen bond synthons and recognition modes. By using the moderate to weak association between pyridine amide and cyclic lactam coformers (Bolla & Nangia, 2015 ▸), a successful ternary combination of ACZ, NAM and 2HP (ACZ–NAM–2HP, 1:1:1) was then derived from an analysis of the binary cocrystals. The molecules are classified as: **A** = ACZ; **B** = pyridine carboxamides, NAM, PAM; and **C** = cyclic lactams: VLM, CPR; *syn* amides: 2HP, MeHP, OMeHP (see Fig. 2 ▸).

## 2. Experimental   

All the coformers used in this study were purchased from Sigma-Aldrich, India. All chemicals are of analytical and chromatographic grade. Acetazolamide was purchased from Yarrow Chemicals, Mumbai, India, and its purity was confirmed by NMR and DSC.

### 2.1. ACZ–NAM (1:1)   

ACZ (100 mg, 0.45 mmol) and NAM (54 mg, 0.45 mmol) were ground well in a mortar and pestle for 20–30 min by adding 4–5 drops of EtOAc. The ground material was kept for crystallization from a solvent mixture of EtOAc and THF (5 ml) as well as in individual solvents in a 25 ml conical flask at room temperature. Good quality crystals were harvested at ambient condition after a week; m.p. 180°C.

### 2.2. ACZ–VLM (1:2)   

ACZ (100 mg, 0.45 mmol) and VLM (45 mg, 0.45 mmol) were taken in a 1:1 ratio and ground well in a mortar and pestle for 20–30 min by adding 4–5 drops of EtOAc. The ground material was kept for crystallization in EtOAc (5 ml) at room temperature. Good quality crystals were harvested at ambient conditions after a week. Even though the components were taken in an equal molar ratio, the product crystallized in a 1:2 ratio from solution; m.p. 93°C.

### 2.3. ACZ–CPR hydrate (1:1:1)   

ACZ (100 mg, 0.45 mmol) and CPR (50 mg, 0.45 mmol) were taken in a 1:1 ratio and ground well in a mortar and pestle for 20–30 min by adding 4–7 drops of EtOAc. The ground material was kept for crystallization from a solvent mixture of EtOAc and THF (5 ml) as well as individual solvents at room temperature. The ground material crystallized from solution as a hydrate after one week; m.p. 80°C. Solvents used here are analytically pure and crystallization was carried out at room temperature (*ca* 30°C) in an open evaporation flask, which gave the cocrystal hydrate product.

### 2.4. ACZ–2HP (1:2)   

ACZ (100 mg, 0.45 mmol) and 2HP (42 mg, 0.45 mmol) were taken in a 1:1 ratio and ground well in a mortar and pestle for 20–30 min by adding 4–7 drops of EtOAc. The ground material was kept for crystallization from a solvent mixture of EtOAc and THF (5 ml) as well as individual solvents at room temperature. Good quality crystals were harvested at ambient conditions after a week. The ground material crystallized from solution in a 1:2 ratio; m.p. 160°C.

### 2.5. ACZ–2HP (1:1)   

ACZ (100 mg, 0.45 mmol) and 2HP (42 mg, 0.45 mmol) were ground well in a mortar and pestle for 20–30 min by adding 4–7 drops of EtOAc in the presence of NAM or INA to obtain a ternary system. Even though the attempts to obtain an ACZ binary cocrystal with isonicotinamide (INA) were not successful, experiments were carried out to obtain a ternary ACZ–INA–2HP adduct in a trial attempt. A binary product ACZ–2HP (1:1) was obtained. A unit cell check of randomly selected crystals showed that the majority are ACZ–2HP (1:1), while a few crystals had 1:2 stoichiometry. The ground material of 1:1 stoichiometry was kept for crystallization from a solvent mixture of EtOAc and THF (5 ml) as well as individual solvents at room temperature. Good quality crystals were harvested at ambient condition after a week; m.p. 180°C.

### 2.6. ACZ–MeHP (1:1)   

ACZ (100 mg, 0.45 mmol) and MeHP (49 mg, 0.45 mmol) were ground well in a mortar and pestle for 20–30 min. The ground material was kept for crystallization in 5 ml of EtOAc at room temperature to obtain good quality single crystals at ambient conditions after 1 week; m.p. 130°C.

### 2.7. ACZ–OMeHP hydrate (1:1:1)   

ACZ (100 mg, 0.45 mmol) and OMeHP (56 mg, 0.45 mmol) were ground well in a mortar and pestle for 25 min with a few drops of EtOAc added. The ground material was kept for crystallization in 5 ml of EtOAc and THF mixture or in the individual solvents at room temperature to give good quality single crystals after 4–5 d. The product crystallized as a monohydrate; m.p. 90°C.

### 2.8. ACZ–NAM–2HP (1:1:1)   

ACZ (100 mg, 0.45 mmol), NAM (54 mg, 0.45 mmol) and 2HP (42 mg, 0.45 mmol) were ground well in a mortar and pestle for 20–30 min by adding 4–7 drops of EtOAc. The ground material was kept for crystallization from a solvent mixture of 5 ml EtOAc and THF as well as the individual solvents at room temperature to give good quality single crystals of the ternary adduct after 5–6 days. A few crystals of binary products ACZ−NAM and ACZ−2HP (1:1, 1:2) were also observed in the crystallization flask concomitantly based on a unit cell check. Single crystal data were collected of the ternary product by manual separation of their different morphology crystals as a plate and a block; m.p. 125°C.

### 2.9. Single-crystal X-ray diffraction   

A single crystal was mounted on the goniometer of an Oxford Diffraction Gemini X-ray diffractometer equipped with Cu *K*α radiation source (λ = 1.54184 Å) at 298 K. Data reduction was performed using *CrysAlisPro* 171.33.55 software (Oxford Diffraction, 2008 ▸). The crystal structure was solved and refined using *Olex*2-1.0 (Dolomanov *et al.*, 2009 ▸) with anisotropic displacement parameters for non-H atoms. H atoms were experimentally located through the difference-Fourier electron density maps in all crystal structures. Data were reduced by *SAINT-Plus* (Bruker, 1998 ▸) and further continued with *SHELXTL* (Sheldrick, 2008 ▸). A check of the final CIF file with *PLATON* (Spek, 2009 ▸) did not show any missed symmetry. *X-Seed* (Barbour, 2001 ▸) was used to prepare the figures and packing diagrams. The crystallographic parameters of all the cocrystals are summarized in Table 1 ▸ and hydrogen-bond distances are listed in Table S1. CIF files are deposited at CCDC Nos. 1436978–1436985. Single-crystal X-ray diffraction data were also collected at 298 K on a Bruker SMART APEX-1 CCD area-detector system equipped with a graphite monochromator Mo *K*α fine-focus sealed tube (λ = 0.71073 Å) operating at 1500 power, 40 kV, 30 mA. The frames were integrated by *SAINT* (Bruker, 1998 ▸) software using a narrow-frame integration algorithm. Data were corrected for absorption effects using the multi-scan method (*SADABS*; Bruker, 1998 ▸). The structures were solved and refined using *SHELXTL* (Sheldrick, 2008 ▸).

### 2.10. X-ray powder diffraction   

Bulk samples were analyzed by X-ray powder diffraction on a Bruker AXS D8 diffractometer (Bruker-AXS, Karlsruhe, Germany). Experimental conditions: Cu *K*α radiation (λ = 1.54056 Å), 40 kV, 30 mA, scanning interval 5–50° 2θ at a scan rate of 1° min^−1^, time per step 0.5 s.

## 3. Results and discussion   

### 3.1. ACZ polymorphs and reported binary adducts   

**Figure 3:**
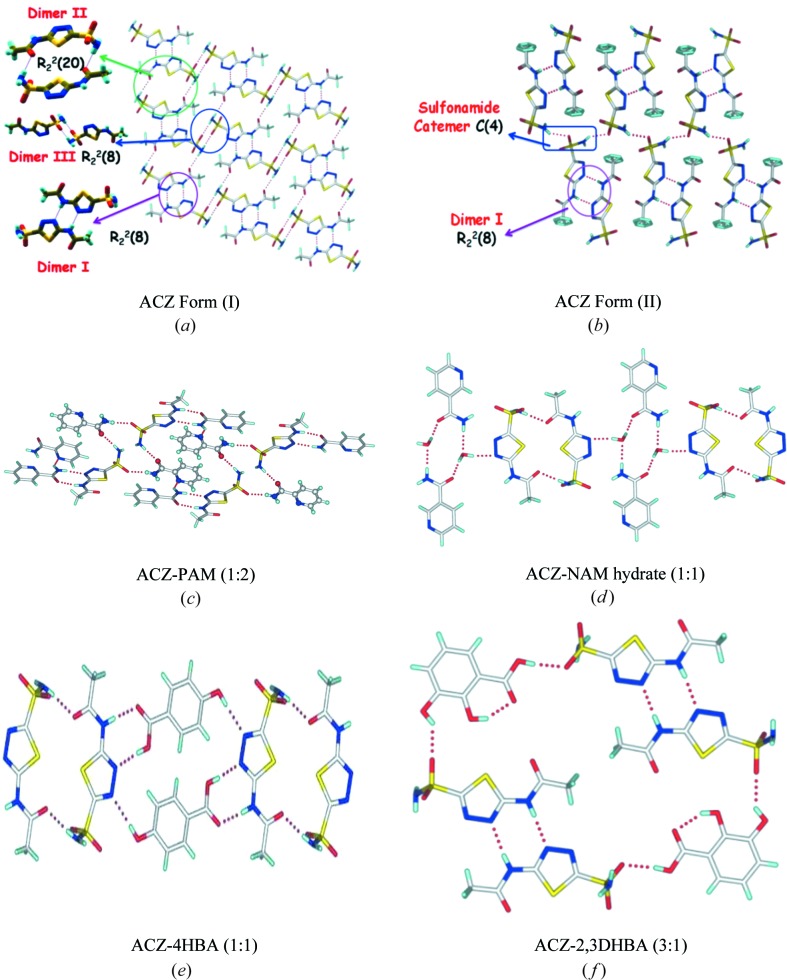
(*a*), (*b*) Supramolecular synthons in ACZ polymorphs Form (I) and (II). (*c*), (*d*), (*e*), (*f*) Binary cocrystals of ACZ with picolinamide, nicotinamide, 4-hydroxybenzoic acid and 2,3-dihydroxybenzoic acid.

Acetazolamide (ACZ, Fig. 2 ▸) consists of a primary sulfonamide group, thiadiazole heterocycle and acetamide groups. Both the sulfonamide and acetamido groups are sites for hydrogen bonding with complementary coformers listed under B and C. Crystal structures of two polymorphs (I) and (II) of ACZ are reported (Umeda *et al.*, 1985 ▸). Form (I) exhibits multiple ring synthons such as N—H⋯N homodimers 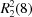, sulfonamide dimer 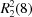, sulfon­amide–amide macrocycle ring 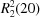 (Fig. 3 ▸ *a*) (Etter, 1990 ▸; Bernstein *et al.*, 1995 ▸). Form (II) comprises sulfonamide catemer chains *C*(4) as well as amide–thiadiazole N—H⋯N ring motif 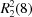 (Fig. 3 ▸ *b*). The hydrogen-bond motifs present in the reported cocrystals of ACZ (Baraldi *et al.*, 2009 ▸; Arenas-García *et al.*, 2010 ▸, 2012 ▸; Ferrer *et al.*, 1989 ▸, 1990 ▸; Hartmann & Vahrenkamp, 1991 ▸) with 4-hydroxybenzoic acid (ACZ–4HBA; 1:1), a hydrate with nicotinamide (ACZ–NAM hydrate; 1:1:1), picolinamide (ACZ–PAM; 1:2) and 2,3-dihydroxy benzoic acid (ACZ–2,3DHBA; 3:1) are displayed in Figs. 3 ▸(*c*)–(*f*). The fact that the synthons in cocrystal structures are quite different from those in the two polymorphs of ACZ means that the coformer functional groups are able to disrupt the self-association to give stronger and newer motifs in the binary complexes. This is a positive indication for successful cocrystallization.

The crystal structures and supramolecular synthons of binary systems with a few pharmaceutically acceptable coformers are discussed to understand the hydrogen bonding and stoichiometry in self-assembly: ACZ–NAM (1:1), ACZ–VLM (1:2), ACZ–CPR hydrate (1:1:1), ACZ–2HP (1:1 and 1:2), ACZ–MeHP (1:1) ACZ–OMeHP hydrate (1:1:1). The designed assembly of a ternary cocrystal ACZ–NAM–2HP (1:1:1) is as such rare for drug molecules.

### 3.2. Crystal structures of binary cocrystals   

The crystal structure parameters are summarized in Table 1 ▸ and hydrogen-bond parameters in Table S1 of the supporting information. The synthons and molecular packing of binary cocrystals is presented first and then the build up to the ternary system is described.

#### 3.2.1. ACZ–NAM (1:1)   

**Figure 4:**
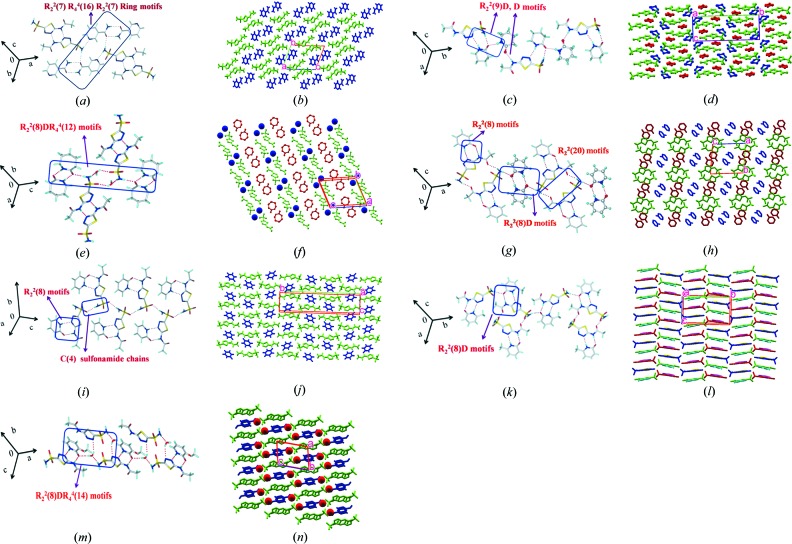
Supramolecular synthons and molecular packing in ACZ binary systems. (*a*), (*b*) ACZ–NAM (1:1) displays ACZ sulfonamide N—H⋯O and N—H⋯N hydrogen bonds with NAM amide dimers and pyridine N motifs. (*c*), (*d*) ACZ–VLM (1:2) where one VLM forms a hydrogen bond with the amidediazole–amide synthon and the second VLM connects such heterodimer units. (*e*), (*f*) CPR homodimers interact with sulfonamide hydrate dimer motifs *via* N—H⋯O and O—H⋯O hydrogen bonds. (*g*), (*h)* ACZ–2HP (1:2) shows one equivalent of 2HP to make the heterodimer which is connected by the second 2HP homodimers and sulfonamide N—H⋯O chain. (*i*), (*j*) Two-dimensional packing in ACZ–2HP (1:1) makes the binary heterosynthon similar to the previous structure as well as the sulfonamide *C*(4) catemer. (*k*), (*l*) ACZ–MeHP (1:1) is similar to that of ACZ–2HP (1:1). (*m*), (*n*) Water molecules are present in the crystal lattice of ACZ–OMeHP hydrate (1:1:1) which connect the binary components of aminodiazole–amide. H atoms are removed in a few diagrams for clarity.

The cocrystal structure (space group 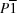) contains N—H⋯N homodimers 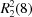 of ACZ, similar to those observed in polymorph (II). However, the sulfonamide dimer of ACZ is replaced by N—H⋯O and N—H⋯N hydrogen bonds to nicotinamide. Two ACZ and two NAM molecules form a tetramer ring motif 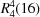 *via* N—H⋯O and N—H⋯N hydrogen bonds (Fig. 4 ▸ *a*). The overall structure has a layered two-dimensional pattern (Fig. 4 ▸ *b*).

#### 3.2.2. ACZ–VLM (1:2)   

In the crystal structure (*P*2_1_/*c*) VLM forms a 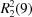 motif of sulfonamide, thiadiazole and amide groups (Fig. 4 ▸ *c*). A second equivalent of VLM connects such heterosynthon units *via* N—H⋯O hydrogen bonds. Such one-dimensional chains extended parallel to the *c*-axis *via* N—H⋯O hydrogen bonds in a two-dimensional array (Fig. 4 ▸ *d*). The inclusion of a second VLM in the cocrystal structure suggested that if this latter molecule could be replaced by a different amide, then a ternary system will result. In other words, the binary system has a tendency to include a third partner from solution. The same phenomenon is observed in the next structure.

#### 3.2.3. ACZ–CPR hydrate (1:1:1)   

The ground material of ACZ and CPR in a 1:1 ratio was crystallized as a hydrate (1:1:1) in space group 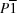. CPR homodimers 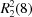 are sandwiched between the SO_2_NH_2_ and water molecules in a 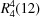 ring motif (Fig. 4 ▸ *e*). Such discrete clusters extend *via* the water O—H donor (Fig. 4 ▸ *f*). Even though the water is serendipitously included, it makes three components in the crystal lattice.

#### 3.2.4. ACZ–2HP (1:2)   

The ground product of ACZ–2HP (1:2) was solved in space group 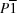. One equivalent of 2HP breaks the strong N—H⋯N homodimer 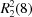 between ACZ molecules and forms an amide(2HP)–imino (ACZ) heterosynthon. The sulfonamide–carboxamide dimer motif 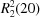 previously noted in ACZ Form (I) (Fig. 3 ▸ *a*) is present in this binary system (Fig. 4 ▸ *g*). The second equivalent of 2HP homodimers connect ACZ−2HP units *via* N—H⋯O hydrogen bonds to give the 1:2 composition (Fig. 4 ▸ *h*). Again, the 2HP dimers could be replaced by a structural mimic to give a ternary system.

#### 3.2.5. ACZ–2HP (1:1)   

The cyclic ring motifs, such as the N—H⋯N dimer of ACZ polymorphs (Figs. 3 ▸ *a* and *b*), are interrupted in the presence of 2HP to give an amide–iminodiazole 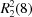 motif (Fig. 4 ▸ *i*), similar to the previous structure. The extended motifs *via* N—H⋯O catemer chain *C*(4) in this structure (Fig. 4 ▸ *j*) obviate the need for the 2HP dimer noted in the 1:2 structure (Fig. 4 ▸ *g*). This binary structure suggests that 2HP should be a good partner for ternary assembly because the crystal structure is heavily disturbed compared with the ACZ structure, as well as other cocrystals. Moreover, both 1:1 and 1:2 combinations were routinely observed. A strong heterodimer between the two components is a prerequisite for ternary assembly (Aakeröy *et al.*, 2001 ▸; Aakeröy & Salmon, 2005 ▸). The presence of the symmetry-independent 2HP dimer in the 1:2 structure appears to be optional and it could be replaced by another component of similar size, shape and hydrogen-bonding groups to yield a ternary cocrystal. We note that there is a similar NAM amide dimer in the ACZ–NAM (1:1) structure (Fig. 4 ▸ *a*) and this gives a logical lead towards the ternary combination.

#### 3.2.6. ACZ–MeHP (1:1)   

The centrosymmetric 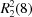 dimers of ACZ−MeHP are formed with N—H⋯O and N—H⋯N bonds between aminodiazole–amide groups (Fig. 4 ▸ *k*), which is similar to the heterosynthon in ACZ–2HP. The sulfonamide N—H donors connect molecules to make extended arrays (Fig. 4 ▸ *l*).

#### 3.2.7. ACZ–OMeHP hydrate (1:1:1)   

The ground product of ACZ and OMeHP in a 1:1 ratio crystallized as a hydrate (1:1:1) in space group 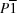. The dimer of ACZ diazole and OMeHP amide in ring motif 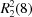 (Fig. 3 ▸ *a*) and water molecules connect such units (Fig. 4 ▸ *m*) in the inter-layer region (Fig. 4 ▸ *n*). It appears that the inclusion of water was mandated as a spacer between the ACZ−OMeHP dimer units to accommodate the OMe group. This means that the bonding between ACZ and 2HP is strong enough to override steric groups which were overcome by the inclusion of a water molecule in the binary cocrystal.

The above crystal structures are described in the natural sequence of crystallization being carried out and the experimental results analyzed.

### 3.3. Crystal structure of ternary cocrystal ACZ–NAM–2HP (1:1:1)   

**Figure 5:**
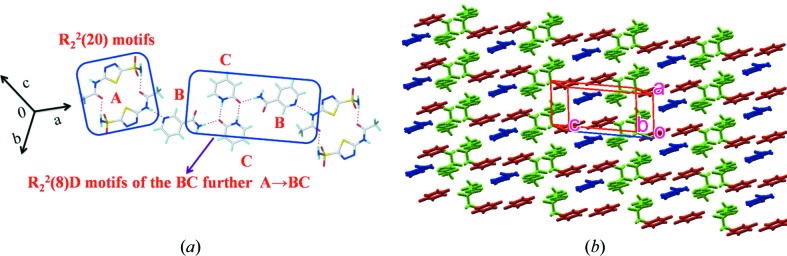
(*a*) Synthons in ternary cocrystal ACZ–NAM–2HP (1:1:1). The macrocycle ring motif 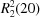 of ACZ are novel to the ternary structure and NAM further extends these units with hydrogen bonding to the dimers of 2HP. (*b*) Two-dimensional packing of the ternary cocrystal shows that ACZ molecules (green) are separated by NAM and 2HP (blue, red).

After screening several binary combinations and their crystal structures, we have decided to replace the second equivalent of the coformer in ACZ−2HP (1:2) with NAM, given that nicotinamide can form an amide 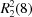 dimer similar to 2HP. Moreover such dimers are present in ACZ–NAM (1:1). Grinding of ACZ, NAM and 2HP in an equimolar ratio and recrystallization of the crystalline product gave the ternary cocrystal ACZ–NAM–2HP, as confirmed by single-crystal X-ray diffraction (1:1:1 stoichiometry). The ternary cocrystal structure has resemblances with the binary structure ACZ–2HP (1:2) as expected. Hydrogen bonds between the sulfonamide NH and acetamide C=O groups of ACZ result in dimer pairs 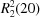 (Fig. 5 ▸ *a*), which were noted previously in polymorph (I) of ACZ as well as in ACZ−2HP (1:2). The 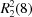 dimers of 2HP are also present here. The link between these ring motifs is that the amide NH of ACZ bonds to the pyridine N of NAM and the NH of NAM is bonded to the 2HP amide dimer. Thus, while the **A** and **C** dimers are repeating motifs, the linkage through the **B** molecule is somewhat different in the ternary structure compared with the previous binary cocrystals. Propagation of the centrosymmetric motifs *via* N—H⋯O and N—H⋯N hydrogen bonds is shown in Fig. 5 ▸(*b*). There is considerable ‘carry over’ of synthons from the binary to the ternary cocrystal, yet there are unexpected motifs as well. Overall, the element of design and crystal engineering appears to be a consistent thread in this family of structures.

### 3.4. Supramoleculer synthons in this study   

**Figure 6:**
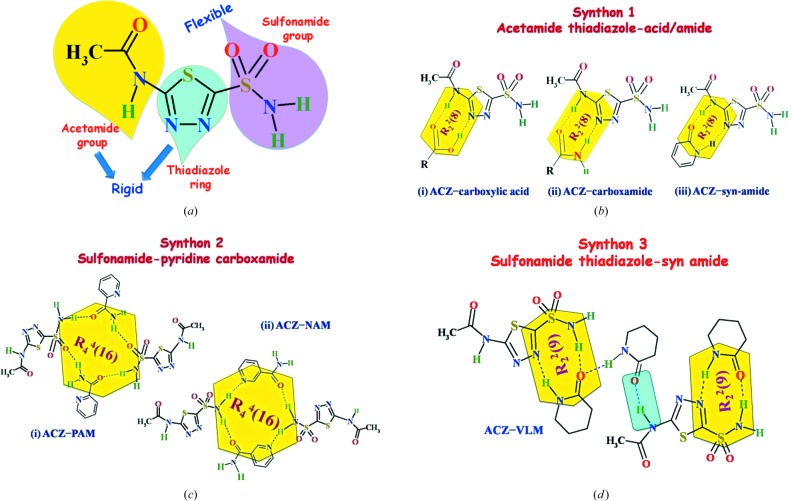
Hydrogen-bonding synthons of ACZ observed in this study. (*a*) Molecular diagram showing the hydrogen bonding groups as rigid or flexible (according to Arenas-García *et al.*, 2010 ▸, 2012 ▸). (*b*) Synthon 1 between the acetamide and thiadiazole ring of ACZ with carboxylic acid, carboxamide, *syn*-amide, respectively. (*c*) Synthon 2 between the sulfonamide group of ACZ and pyridine carboxamides, *e.g.* NAM, PAM, to give large ring motifs. (*d*) Synthon 3 connects sulfonamide, thiadiazole N and lactam conformer **C**. The graph-set notations of ring motifs are given for identification.

The three hydrogen bonding sites in ACZ are acetamide (donor–acceptor), sulfonamide group (donor–acceptor) and thiadiazole ring (acceptor only) (Fig. 6 ▸ *a*). The sulfonamide is flexible (Arenas-García *et al.*, 2010 ▸, 2012 ▸) while the other two moieties, acetamide and thiadiazole, are rigid functional groups for hydrogen bonding. The main synthons observed in different cocrystals are displayed in Figs. 6 ▸(*b*)–(*d*).

**Figure 7:**
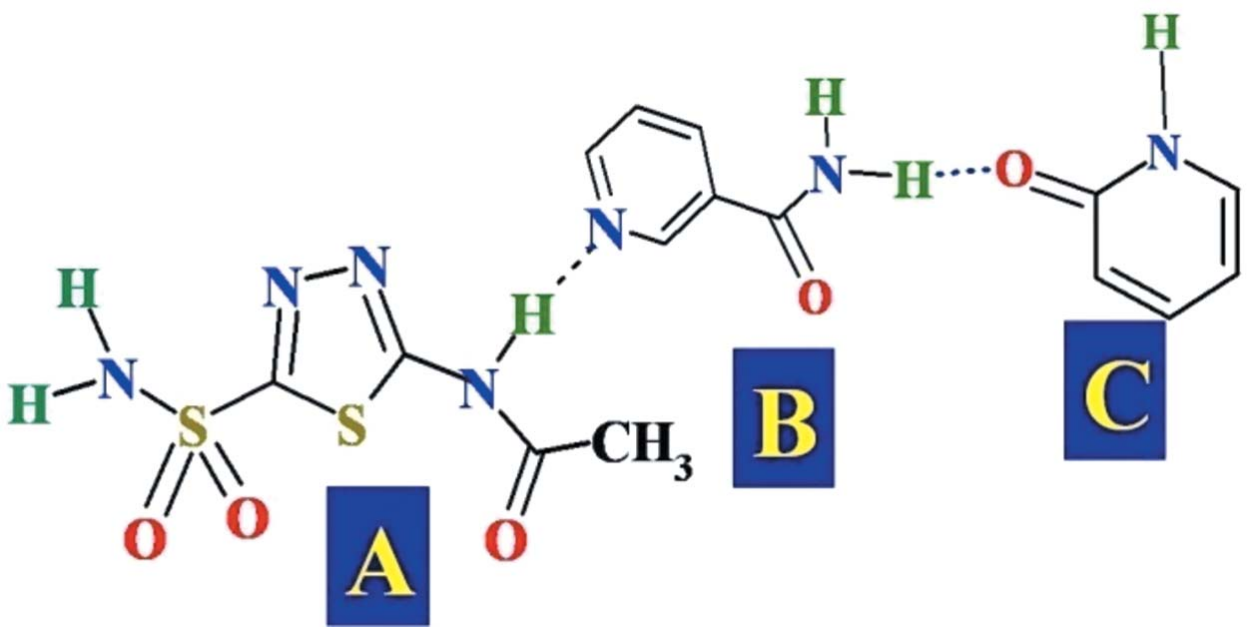
LSAM in the ternary assembly ACZ–NAM–2HP.

The main stream of our approach and objective was to understand the long-range synthon Aufbau modules (LSAM; Ganguly & Desiraju, 2010 ▸; Mukherjee *et al.*, 2014*a* ▸,*b* ▸) in the ternary cocrystals (Bolla & Nangia, 2015 ▸). However, because we were successful in crystallizing only a single ternary structure in this family, a supramolecular build-up or LSAM model for the ternary assembly of ACZ is difficult to analyze due to insufficient data. The ternary cocrystal suggests the ACZ amide bonds with NAM pyridine *via* N—H⋯N and the CONH_2_ donor of NAM connects to the pyridone *via* N—H⋯O to give the ternary adduct (Fig. 7 ▸).

### 3.5. CSD analysis of heterosynthons in this study   

**Figure 8:**
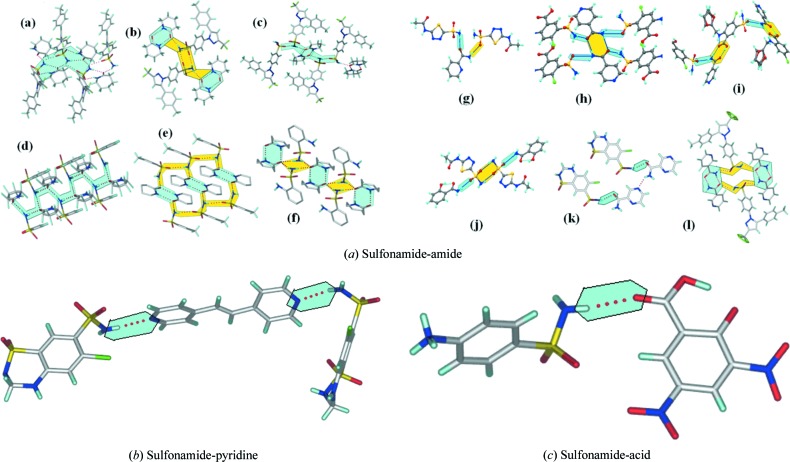
Different types of (*a*) sulfonamide–amide, (*b*) sulfonamide−pyridine and (*c*) sulfonamide−acid supramolecular synthons.

A search of the Cambridge Structural Database (CSD Version 5.36, May 2015 update) was carried out to tabulate the reported supramolecular synthons of the sulfonamide functional group observed in this study (after eliminating hydrates, solvates, salts and duplicates) with those reported in previous structures. Relatively few hits are obtained on the sulfonamide functional group bonding to amide (25 hits), pyridine (4 hits) and carboxylic acids (3 hits); see Table 2 ▸ and Table S2. The present study therefore is an early result on cocrystals of a sulfonamide drug with pyridine amide and lactam conformers (GRAS-type molecules; Bolla & Nangia, 2015 ▸). The synthons extracted from the CSD for a sulfonamide bonding to an amide or pyridine group are displayed in Fig. 8 ▸. Analogous to the carboxamide group (Nangia, 2010 ▸), the occurrence of SO_2_NH_2_ bonding with an amide group is much more likely than to a pyridine (six times) due to the stronger amide acceptor nature.

## 4. Conclusions   

The first study of sulfonamide drug cocrystals with amide coformers is described leading to a ternary drug cocrystal. A library of supramolecular synthons was derived from binary adducts of ACZ with pyridine amide and lactam coformers (**A**–**B** and **A**–**C** cocrystals). There is competition and interplay of the hydrogen bonding functional groups during binary cocrystallization. The binary results suggest that the *syn* amides form reliable synthons to afford cocrystals with a sulfonamide drug. Using ACZ–NAM and ACZ–2HP cocrystals as leads, a ternary assembly was designed to give the 3-component cocrystal ACZ–NAM–2HP (1:1:1). Mechanochemistry, or grinding with solvent added, is necessary to hydrogen bond the components in the ternary adduct, which was then recrystallized to produce single crystals for X-ray diffraction. This is the first crystal structure report of a sulfa drug ternary cocrystal.

## Supplementary Material

Crystal structure: contains datablock(s) global, ACZ2HP11, ACZ2HP12, ACZCPRH111, ACZDMSO, ACZMeHP11, ACZNAM11, ACZNAM2HP111, ACZOMeHPH111, ACZVLM12. DOI: 10.1107/S2052252516000543/ed5007sup1.cif

Structure factors: contains datablock(s) ACZ2HP11. DOI: 10.1107/S2052252516000543/ed5007ACZ2HP11sup2.hkl

Structure factors: contains datablock(s) ACZHP21. DOI: 10.1107/S2052252516000543/ed5007ACZ2HP12sup3.hkl

Structure factors: contains datablock(s) ACZCPRH111. DOI: 10.1107/S2052252516000543/ed5007ACZCPRH111sup4.hkl

Structure factors: contains datablock(s) ACZDMSO. DOI: 10.1107/S2052252516000543/ed5007ACZDMSOsup5.hkl

Structure factors: contains datablock(s) ACZMeHP11. DOI: 10.1107/S2052252516000543/ed5007ACZMeHP11sup6.hkl

Structure factors: contains datablock(s) ACZNAM11. DOI: 10.1107/S2052252516000543/ed5007ACZNAM11sup7.hkl

Structure factors: contains datablock(s) ACZNAM2HP111. DOI: 10.1107/S2052252516000543/ed5007ACZNAM2HP111sup8.hkl

Structure factors: contains datablock(s) ACZOMeHPH111. DOI: 10.1107/S2052252516000543/ed5007ACZOMeHPH111sup9.hkl

Structure factors: contains datablock(s) ACZVLM12. DOI: 10.1107/S2052252516000543/ed5007ACZVLM12sup10.hkl

Supporting tables. DOI: 10.1107/S2052252516000543/ed5007sup11.pdf

CCDC references: 1436985, 1436984, 1436983, 1451431, 1436982, 1436980, 1436981, 1436979, 1436978

## Figures and Tables

**Table 1:** Crystallographic parameters of cocrystals

	ACZ–NAM (1:1)	ACZ–VLM (1:2)	ACZ–CPR hydrate (1:1:1)	ACZ–2HP (1:2)	ACZ–2HP (1:1)	ACZ–MeHP (1:1)	ACZ–OMeHP hydrate (1:1:1)	ACZ–NAM–2HP (1:1:1)
Empirical formula	C_4_H_6_N_4_O_3_S_2_·C_6_H_6_N_2_O	C_4_H_6_N_4_O_3_S_2_·2C_5_H_9_NO	C_4_H_6_N_4_O_3_S_2_·C_6_H_11_NO·H_2_O	C_4_H_6_N_4_O_3_S_2_·2C_5_H_5_NO	C_4_H_6_N_4_O_3_S_2_·C_5_H_5_NO	C_4_H_6_N_4_O_3_S_2_·C_6_H_7_NO	C_4_H_6_N_4_O_3_S_2_·C_6_H_7_NO_2_·H_2_O	C_4_H_6_N_4_O_3_S_2_·C_6_H_6_N_2_O·C_5_H_5_NO
Formula weight	344.38	420.51	353.42	412.45	317.35	994.12	775.22 (9)	439.48
Crystal system	Triclinic	Monoclinic	Triclinic	Triclinic	Monoclinic	Monoclinic	Triclinic	Triclinic
Space group	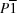	*P*2_1_/*c*	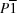	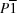	*P*2_1_/*n*	*Pc*	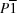	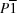
*T* (K)	298	298	298	298	298	298	298	298
*a* (Å)	5.1477 (8)	9.66166 (19)	4.9969 (2)	6.8501 (3)	4.9138 (4)	11.3972 (7)	7.7872 (6)	7.0347 (3)
*b* (Å)	10.8147 (14)	23.4685 (4)	11.6983 (6)	11.3563 (6)	33.192 (3)	18.1641 (3)	10.2130 (7)	10.2539 (7)
*c* (Å)	14.2604 (16)	8.84352 (17)	14.6244 (8)	12.3387 (8)	8.3659 (7)	10.338 (3)	10.2464 (7)	13.7934 (9)
α (°)	69.797 (11)	90	70.868 (5)	82.288 (5)	90	90	88.192 (5)	81.685 (6)
β (°)	85.463 (12)	100.773 (1)	81.892 (4)	81.856 (4)	99.520 (1)	97.046 (16)	76.587 (6)	83.028 (5)
γ (°)	81.889 (12)	90	80.262 (4)	75.804 (4)	90	90	88.192 (5)	88.283 (5)
*V* (Å^3^)	737.20 (18)	1969.88 (6)	792.65 (7)	916.19 (9)	1345.7 (2)	2124.0 (6)	775.22 (10)	977.14 (5)
*D_x_* (g cm^−3^)	1.55	1.42	1.48	1.49	1.56	1.55	1.56	1.49
*Z*	2	4	2	2	4	6	2	2
*R*_1_ [*I* > 2σ(*I*)]	0.0631	0.0427	0.0378	0.0647	0.0378	0.0397	0.0492	0.0612
*wR*_2_ (all)	0.1944	3507	2792	0.1710	0.0984	4590	0.1408	0.1789
Goodness-of-fit	1.005	1.064	1.078	1.061	1.000	1.020	1.057	1.083
X-ray diffracto­meter	Oxford CCD	Oxford CCD	Oxford CCD	Oxford CCD	Bruker APEX	Oxford CCD	Oxford CCD	Oxford CCD

**Table 2:** Frequency of sulfonamide synthons in the CSD

Supramolecular synthons of SO_2_NH_2_ group in crystal structure adducts	Number of hits
Sulfonamide–amide	25
Sulfonamide–pyridine	4
Sulfonamide–acid	3
