# Supplementary material for: Binary and ternary cocrystals of sulfa drug acetazolamide with pyridine carboxamides and cyclic amides
Source: IUCrJ. 2016 Feb 25;3(Pt 2):152–60. doi: 10.1107/S2052252516000543 (PMC4775163; doi:10.1107/S2052252516000543)
Supplement: Supplementary file 11 [file m-03-00152-sup11.pdf]

IUCrJ

Volume 3 (2016)

Supporting information for article:

Binary and ternary cocrystals of sulfa drug acetazolamide with pyridine carboxamides and cyclic amides

Geetha Bolla and Ashwini Nangia

Table S1 Hydrogen bond geometry of the crystal structures (neutron normalized) in this paper.

Ccocrystal	Interaction	H···A /Å	D···A /Å	∠D–H···A /º	Symmetry code
ACZ–VLM (1:2)	N3–H3A···O4	1.93	2.789(2)	174	1+x,1/2-y,-1/2+z
	N4–H4A···O5	2.03	2.881(3)	170	1+x,y,z
	N4–H4B···O4	2.21	2.920(3)	159	1+x,y,z
	N5–H5A···O5	2.03	2.873(2)	166	x,-y,1-z
	N6–H6C···N1	2.15	2.999(3)	167	-1+x,y,z
ACZ–CPR hydrate (1:1:1)	N3–H3A···N2	2.13	2.937(2)	176	-x,1-y,-z
	N4–H4D···O4	1.9	2.810(2)	176	1-x,-y,1-z
	N4–H4E···O5	1.96	2.844(3)	164	x,-1+y,z
	N5–H5A···O4	2.13	2.931(3)	176	1-x,1-y,-z
	O5–H5B···O2	2.26	3.020(3)	155	-1+x,1+y,z
	O5–H5B···O2	2.59	3.125(3)	124	1-x,1-y,-z
	O5–H5C···N1	2.41	3.123(3)	167	1-x,1-y,-z
	C4–H4B···O2	2.58	3.516(3)	165	1-x,1-y,-z
	C10–H10B···O3	2.51	3.471(3)	172	----[a]
ACZ–2HP (1:2)	N3–H3A···O5	1.85	2.705(3)	173	1-x,1-y,-z
	N4–H4A···O4	2.01	2.834(3)	172	2-x,1-y,1-z
	N4–H4B···O1	2.09	2.967(3)	172	2-x,2-y,-z
	N5–H5A···O4	1.98	2.822(3)	166	2-x,1-y,1-z
	N6–H6A···N2	2.10	2.954(3)	169	1-x,1-y,-z
	C6–H6···O3	2.51	3.143(4)	126	1-x,1-y,1-z
	C12–H12···O1	2.58	3.479(3)	162	1-x,2-y,-z
	C13–H13···O2	2.58	3.471(4)	160	1-x,2-y,-z
ACZ–2HP (1:1)	N3–H3A···O4	1.86	2.744(2)	177	1+x,y,1+z
	N4–H4D···N1	2.152	2.973(2)	163	-1+x,y,z
	N4–H4E···O3	2.056	2.893(2)	167	1/2+x,1/2-y,1/2+z
	N5–H5A···N2	2.21	2.966(3)	171	-1+x,y,-1+z
	C4–H4B···O1	2.58	3.528(3)	170	-x,-y,1-z
ACZ–MeHP (1:1)	N4–H4D···N5	2.368	2.368(4)	147	----[a]
	N4–H4E···O12	2.133	2.966(5)	178	x,-y+1,+z-1/2
	N8–H6A···O3	2.244	3.141(1)	150	x,-y+1,+z-1/2

	N8–H6B···O5	1.861	2.939(1)	175	x,-y+1,+z-1/2
	N7–H41···O10	1.816	2.674(2)	175	x,-y+1,+z-1/2
	N8–H6B···O7	2.257	2.995(3)	157	x,+y,+z-1
	N11–H11A···O12	1.881	2.740(2)	178	x-1,+y,+z
	N12–H12E···O9	2.708	2.965(1)	103	x,-y,+z+1/2
	N12–H12E···O4	2.311	2.986(2)	156	x,+y,+z+1
	N13–H13A···N6	2.143	2.995(2)	170	x,-y+1,+z+1/2
	N3–H53A···O11	1.964	2.771(2)	170	x+1,+y,+z
ACZ–OMeHP hydrate (1:1:1)	N3–H3A···O5	2.00	2.795(3)	174	2-x,-y,-z
	N4–H4D···O6	2.05	2.844(3)	168	1-x,-y,1-z
	N4–H4E···O4	2.23	3.047(3)	150	----
	N5–H5A···N2	2.03	2.959(3)	176	2-x,-y,-z
	O6–H6A···O5	1.95	2.846(3)	167	----
	O6–H6B···N1	2.34	3.006(4)	149	-1+x,y,z
	C4–H4A···O2	2.49	3.404(4)	160	x,y,-1+z
	C7–H7···O1	2.50	3.426(3)	177	1-x,1-y,-z
ACZ–NAM (1:1)	N3–H3A···N2	2.02	2.873(5)	175	-x,-y,1-z
	N4–H4D···N5	2.13	2.915(6)	165	x,-1+y,z
	N4–H4E···O4	2.11	2.893(5)	165	2-x,1-y,-z
	N6–H6A···O4	2.06	2.916(5)	174	3-x,1-y,-z
	N6–H6B···O2	2.31	3.089(5)	151	----
	C6–H6···O4	2.48	2.805(6)	101	Intramolecular
	C7vH7···O3	2.55	3.438(6)	160	-1+x,1+y,z
	C8–H8···O1	2.55	3.469(7)	168	----
ACZ–NAM–2HP (1:1:1)	N3–H3A···N5	2.05	2.904(3)	176	1-x,1-y,1-z
	N4–H4D···O5	1.89	2.810(3)	168	2-x,-y,1-z
	N4–H4E···O4	2.14	2.954(3)	173	2-x,1-y,-z
	N6–H6B···O1	1.98	2.840(3)	174	-----
	N7–H7A···O1	1.93	2.785(3)	174	1+x,-1+y,z
	C7–H7···N1	2.61	3.238(4)	126	------
	C8–H8···N2	2.62	3.446(4)	149	------
	C9–H9···N2	2.61	3.400(4)	143	1-x,1-y,1-z
	C15–H15···O5	2.52	3.359(4)	151	1-x,-y,1-z

Table S2 REFCODEs of sulfonamide–amide, sulfonamide–pyridine and sulfonamide–acid supramolecular synthons in the CSD.

Sulfonamide−amide (25)				
DATFEL	DATFIP	EGENIP	LIQQEJ	LIQQIN
LIQQIN01	LIQQIN02	LOFLID	NUMGUZ	NUMHAG
NUMHEK	NUMHIO	NUMHOU	NUMHUA	NUMJAI
NUMJEM	NUMJIQ	NUMJOW	NUMJUC	NUMKAJ
NUMKEN	PIRYAS	SANAPY	XAVTEV	YASGOQ02[a]
YASGOQ04[a]				
Sulfonamide−pyridine (4)				
EGEPAJ	POVGAK	POVGEO	POVGIS	
Sulfonamide–acid (3)				
ACIFAT	BOKHAM	LOFLAV		

[a] The same Refcode is repeated twice.
